# A comparative analysis of ENCODE and Cistrome in the context of TF binding signal

**DOI:** 10.1186/s12864-024-10668-6

**Published:** 2024-08-30

**Authors:** Stefano Perna, Pietro Pinoli, Stefano Ceri, Limsoon Wong

**Affiliations:** 1 Lee Kong Chian School of Medicine, Nanyang Technological University, 9 Nanyang Drive, 636921 Singapore, Singapore; 2 Department of Electronics, Information and Bioengineering, Politecnico di Milano, 32 Piazza Leonardo da Vinci, 20133 Milano, Italy; 3 School of Computing, National University of Singapore, 13 Computing Drive, 117417 Singapore, Singapore

**Keywords:** Transcription Factors, Database, SignalValue, ENCODE, Cistrome

## Abstract

**Background:**

With the rise of publicly available genomic data repositories, it is now common for scientists to rely on computational models and preprocessed data, either as control or to discover new knowledge. However, different repositories adhere to the different principles and guidelines, and data processing plays a significant role in the quality of the resulting datasets. Two popular repositories for transcription factor binding sites data - ENCODE and Cistrome - process the same biological samples in alternative ways, and their results are not always consistent. Moreover, the output format of the processing (BED narrowPeak) exposes a feature, the signalValue, which is seldom used in consistency checks, but can offer valuable insight on the quality of the data.

**Results:**

We provide evidence that data points with high signalValue(s) (top 25% of values) are more likely to be consistent between ENCODE and Cistrome in human cell lines K562, GM12878, and HepG2. In addition, we show that filtering according to said high values improves the quality of predictions for a machine learning algorithm that detects transcription factor interactions based only on positional information. Finally, we provide a set of practices and guidelines, based on the signalValue feature, for scientists who wish to compare and merge narrowPeaks from ENCODE and Cistrome.

**Conclusions:**

The signalValue feature is an informative feature that can be effectively used to highlight consistent areas of overlap between different sources of TF binding sites that expose it. Its applicability extends to downstream to positional machine learning algorithms, making it a powerful tool for performance tweaking and data aggregation.

**Supplementary Information:**

The online version contains supplementary material available at 10.1186/s12864-024-10668-6.

## Background

Transcription Factors (TFs) are critical components of the DNA transcription machinery. Their role is to bind their target motifs on TF binding sites and increase or decrease their accessibility. Positional knowledge of Transcription Factor Binding Sites (TFBSs) is critical for understanding, predicting, and acting upon the RNA polymerase machinery. Such knowledge is obtained directly from in vivo Chromatin ImmunoPrecipitation followed by Sequencing (ChIP-Seq) experiments [1]. Data from ChIP-Seq is typically processed, stored, and analysed in the form of large datasets with table-like structures, and computational methods have become increasingly popular for mining ChIP-Seq experiment results.

Several repositories are available that host large quantities of such datasets. The ENCODE database [2] has become a reference for the community in terms of processing, storing, and distribution. Users may freely download hundreds of TFBS datasets from different cellular environments and experimental setups. Data are contributed by the ENCODE Consortium, according to a pipeline designed to maximise consistency between replicates [3]. However, alternative pipelines have been developed to extract different aspect of the biological data. For example, the Cistrome research group [4] collected experimental data from several projects, including but not exclusively ENCODE, and processed them using an in-house pipeline [5]. The goal of Cistrome is to apply an alternative, less strict approach to data filtering and quality control, and thus preserve binding sites with informational content that would otherwise be discarded by stricter quality control. Nevertheless, quality control and analysis pipelines applied to Cistrome datasets need to be robust with respect to a broader set of ChIP-Seq protocols.

**Fig. 1:**
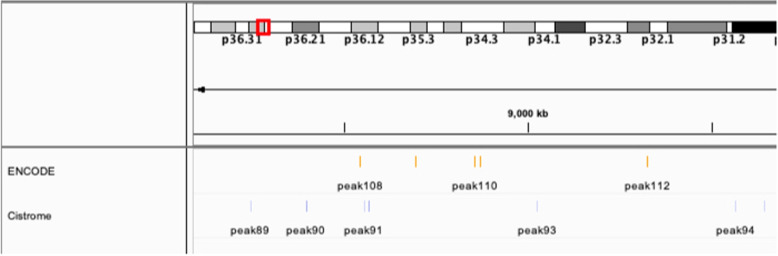
Slice of human chromosome 1, showing ENCODE (orange) and Cistrome (blue) data of the same TF (Nuclear Respiratory Factor 1 (NRF1)) in the same cell line (HepG2). Several ENCODE sites are not replicated by Cistrome, and viceversa

Since ENCODE and Cistrome share a portion of their source data, it seems reasonable to use them interchangeably to feed computational pipelines. For example, overlaps between transcription regions in the two could be considered to be “high confidence” areas to be analysed; furthermore, it is often the case that not a lot of data is available in a single database for a particular biological TF or cell line, so having multiple, compatible sources is desirable. However, data processed by different pipelines are not equivalent. For example, in Fig. 1 we show a small sample of TFBS data from ENCODE and Cistrome pertaining to the same portion of the genome. It is clear that many binding sites are not concordant, neither in quantity nor in position.

One output feature of ChIP-Seq peak callers that is rarely discussed is the signalValue. The signalValue is defined as the average enrichment of a region (cf. https://genome.ucsc.edu/FAQ/FAQformat.html). Enrichment is defined as the number of reads found at a location during an experiment, viz., the higher the signalValue, the more genomic material of interest was found. Binding sites are typically considered in binary terms as either “enriched” or not; however, some algorithms employ signalValue(s) to estimate the strength of binding at a binding site and in the background [6, 7].

In this study, we investigate the overlap between the two databases (ENCODE and Cistrome), and ask if the signalValue feature of narrowPeaks may be used to filter for locations that have a high correspondence. This is not a straightforward question. First, it is not obvious under which parameters should the two data sources be compared, as different applications require different data types and accuracy metrics [8]. Second, data normalisation is of concern: even though both ENCODE and Cistrome take care in ensuring their experiments are reproducible, their datasets are not immediately comparable without processing. Third, it is not clear how to validate the results of such an analysis.

Ultimately, we are interested in deriving sound, validated guidelines for merging and intersecting ENCODE and Cistrome databases. It should be noted that we are interested in mirroring the use case of an investigator who wants to employ publicly available data and cannot or does not want to replicate the peak-calling procedure. As such, we will use the datasets “as-is” and do not perform the peak-calling a second time.

## Results

In this section, we show results obtained in three different cell lines: HepG2, GM12878, and K562. We only report plots for cell line HepG2, as there are more Transcription Factors (TFs) and samples in said cell line, and thus results are more detailed. Plots for the other cell lines are reported in Supplementary Materials, Figures S2 through S7. Results are similar, and will be highlighted in the text for comparison.

### Jaccard Index and Alroy-Forbes coefficient analysis confirms lack of overlap

**Fig. 2:**
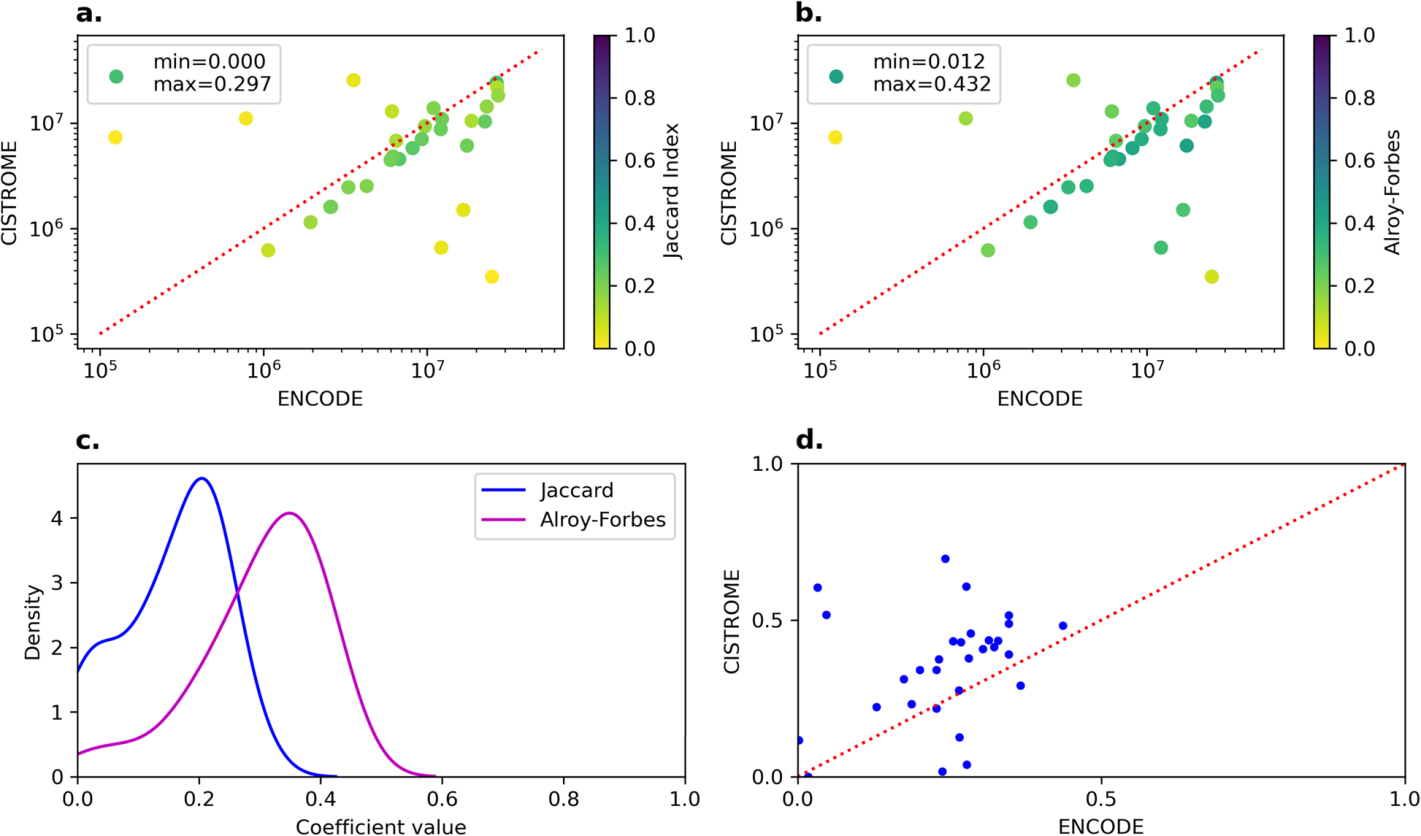
In all graphs, a dot represents a single TF. **a** General JaccardIndex scatterplot for HepG2. *x axis:* number of basepairs occupied by all regions for this TF in ENCODE (log10) *y axis:* number of basepairs occupied by all regions for this TF in Cistrome (log10) *Color:* General JaccardIndex. Darker color represent higher values. **b** Alroy-Forbes coefficient scatterplot for HepG2. *x axis:* number of basepairs occupied by all regions for this TF in ENCODE (log10) *y axis:* number of basepairs occupied by all regions for this TF in Cistrome (log10) *Color:* Alroy-corrected Forbes coefficient. Darker color represent higher values. **c** Distribution of Jaccard Index and Alroy-corrected Forbes coefficient values in HepG2. Note that a value of 1 of the Alroy-Forbes coefficient denotes high-correlation. **d** Conditional Jaccard Index scatterplots on HepG2. *x axis:* Conditional JaccardIndex with respect to ENCODE. *y axis:* Conditional JaccardIndex with respect to Cistrome

In Fig. 2a we report the general Jaccard Indices of ENCODE and Cistrome regions in cell line HepG2.

The minimum and maximum values for the general Jaccard Index in HepG2 are 0.001 and 0.318 respectively (GM12878: 0.021-0.233, K562: 0.007-0.317). This is a clear indication that, at face value, there is little to no overlap of the peak summit windows between ENCODE and Cistrome. Even when we consider the conditional overlaps, the Jaccard Index does not exceed 0.5, stabilizing around the 0.25 to 0.4 range (all cell lines). One might observe that the general Jaccard Index does not tell the full story. Indeed, we expect the Jaccard index to be artificially lower if one dataset has many more regions than the other, even though one of them might be a perfect subset of the other (Fig. 2a and b). Nevertheless, the Alroy-corrected Forbes coefficient also peaks at 0.450 to 0.550 (Fig. 2c, Supplementary Figures S2 and S5), well below the 0.600 threshold, providing stronger evidence to our claim (cf. [9] for a discussion of the range of values of the Alroy-corrected Forbes coefficient).

### Conditional probabilities indicate higher than expected overlap of medium- and high-signal binding sites

**Fig. 3:**
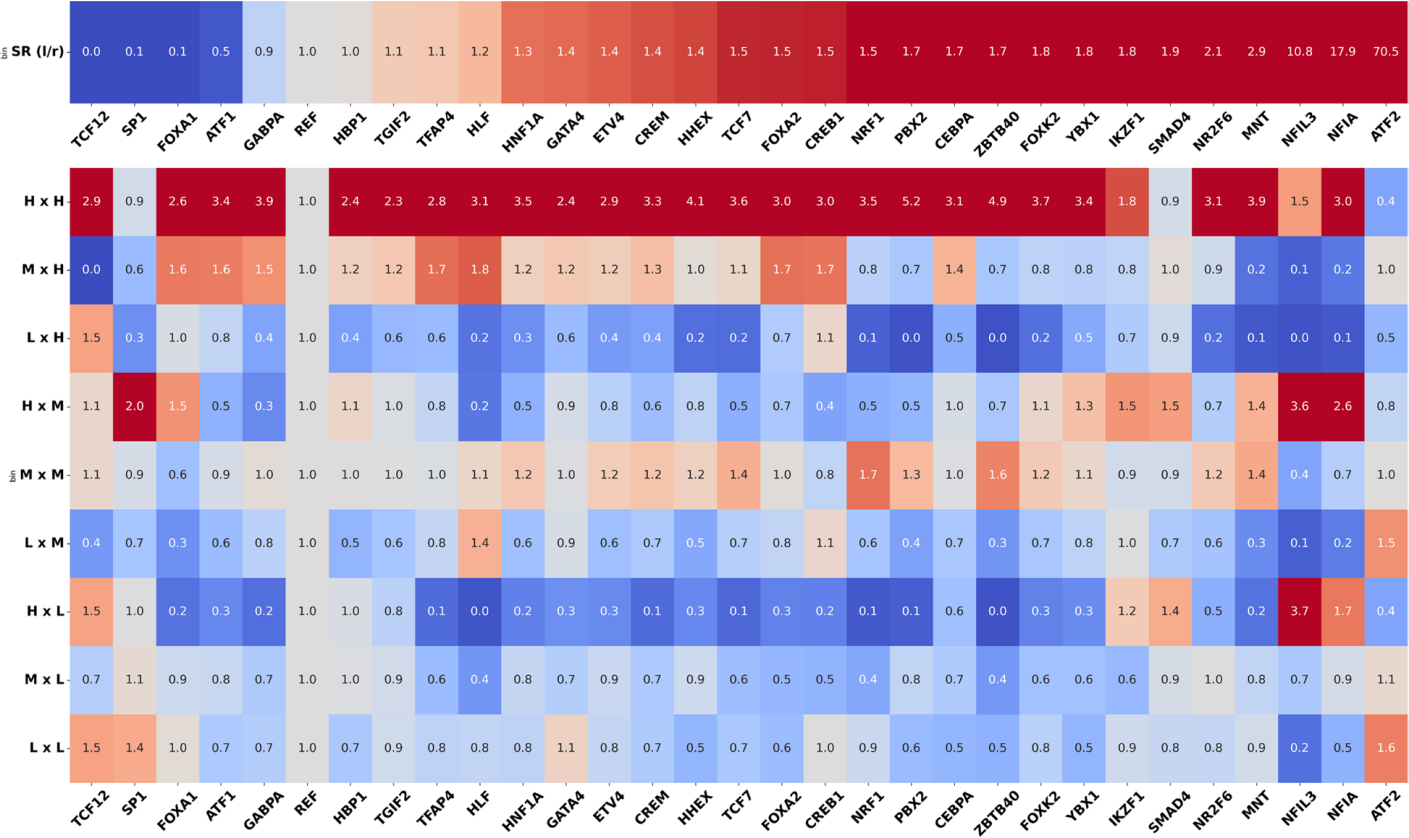
Heatmaps of the signal distribution among joined binding sites in HepG2. **Top row:** size ratio between ENCODE and Cistrome. A size ratio *x* means that the number of binding sites in ENCODE divided by the number of those in Cistrome equals *x* (red: ENCODE has more binding sites, blue: Cistrome has more). **Bottom rows:** for each TF (columns), ratio of joined binding sites that fall into each bin compared to the expected null distribution under independence assumption. *Red:* bin is over-represented compared to the null; *blue:* bins is underrepresented

In spite of this lack of spatial overlap, we can look for a different kind of coherence that considers the signalValue as well. To do so, we investigated whether it is more likely that binding sites with a high signalValue overlap each other. To do so, we plotted the ratio of the observed probability of each of the 9 possible sub-joins based on signal (L matching L, L matching M, and so on) for each of the 30 eligible Transcription Factors (TFs) in HepG2 (Fig. 3, Supplementary Figures S3 and S6), divided by the expected probability of the same under independence assumption. Recall that we binned the distribution of signalValue(s) in each database in 3 baskets (Low, Medium, High) such that the relative ratios are 1:2:1. In other words, P(E=L)=0.25, P(E=M)=0.5, P(E=H)=0.25 (and ditto for Cistrome). Now, if we assume that the probability of two Transcription Factor Binding Sites (TFBSs) matching between databases is independent of the signalValue, we can easily compute the joint probabilities, e.g. P(E=H∧C=H)=0.25∗0.25=0.0625.

We notice that the vast majority of the overlaps fall into the M x M, M x H, H x M, and H x H bins, which are strongly over-represented compared to theoretical distribution (ratio greater than 1, red color). Conversely, most bins that include the L label are underrepresented (ratio less than 1, blue color). Some outliers can be observed, for example Nuclear factor interleukin 3 regulated (NFIL3) in HepG2 or Specificity Protein 1 (SP1) in K562. We note that these (and other) outliers display a large imbalance in the number of binding sites between databases (e.g., ENCODE has 8.6 times the number of Cistrome binding sites in NFIL3, after preprocessing). Despite these outliers, there is a trend for matched binding sites to fall in high signal bins.

Using the above, we can also investigate if the conditional distributions $P(E | C_h)$ (the probability distribution of the signalValue in ENCODE sites that match Cistrome-high sites) and $P(C | E_h)$ (vice versa) are close to the expected values. This is interesting for us because it is direct evidence of whether high signalValue points tend to bind with each other.

**Fig. 4:**
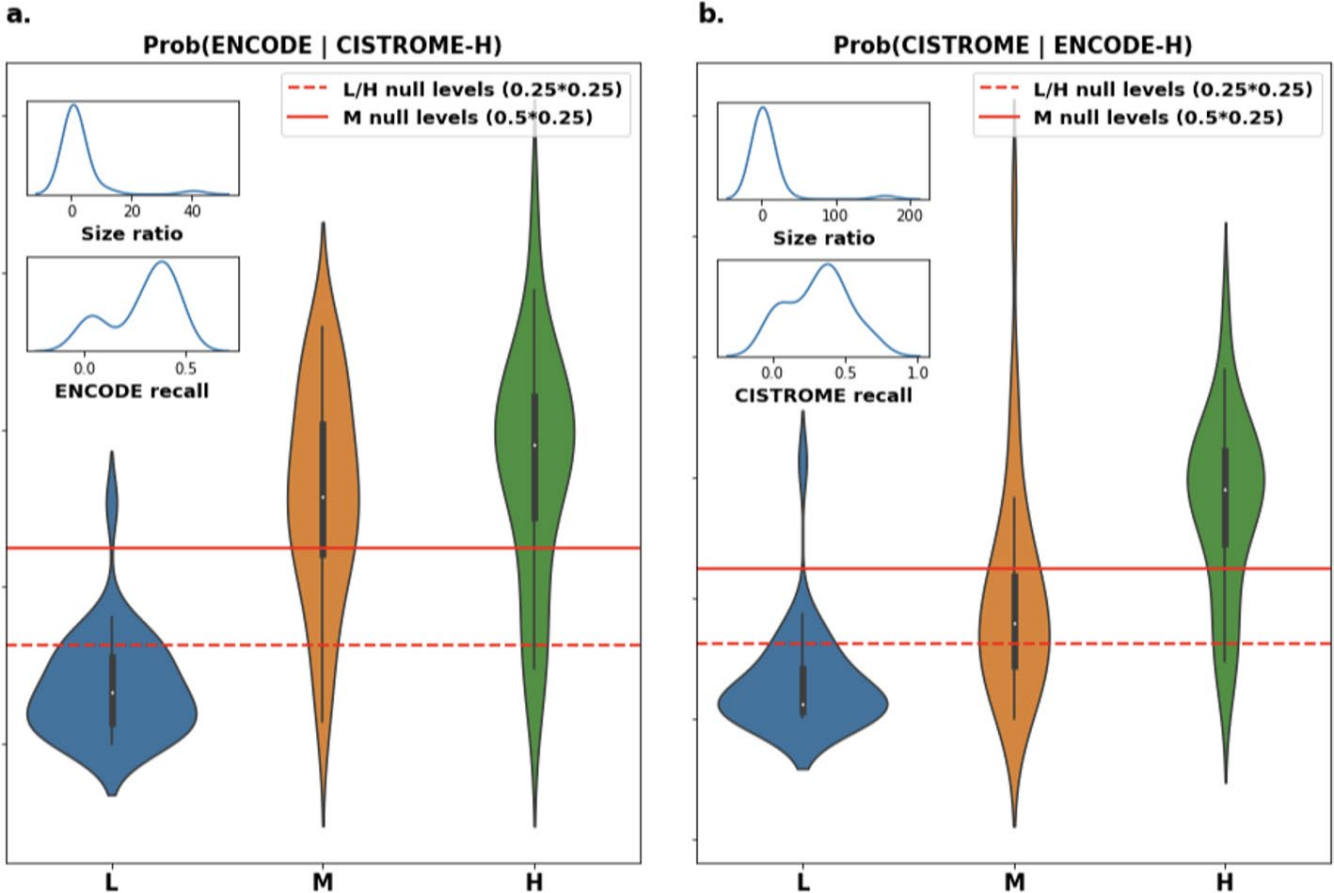
**a** Conditional probability of ENCODE signal when matched with Cistrome-high binding sites. Red Lines: theoretical expected values assuming signal is independent of matching. Inset graphs: top - distribution of the size ratio between ENCODE sites and Cistrome sites among all Transcription Factors (TFs); bottom - distribution of the percentage of ENCODE sites recalled by Cistrome among all Transcription Factors (TFs). **b** Conditional probability of Cistrome signal when matched with ENCODE-high binding sites. Red Lines: theoretical expected values assuming signal is independent of matching. Inset graphs: as left, with reverse databases

In Fig. 4a and b we chart the observed distributions of the conditional probabilities in HepG2, along with the null conditional probabilities (solid and dashed red lines). In supplementary Figures S4 and S7, we report the same for GM12878 and K562 respectively.

We observe in all cases a disproportionate amount of high signal sites matching another high signal site, and a below average number of low signal sites matching a high signal site. This observation, combined with the previous ones, strongly suggests that the independence assumption does not hold for these dataset(s). We also observe that ENCODE-medium has a higher than expected probability of matching with Cistrome-high, while the reverse is not true (consistent across all 3 cell lines). By combining the inset figures for ENCODE recall and Fig. 3 (and Supplementary Figures S4 and S7), we note that this EC-M over-representation is due to Transcription Factors (TFs) that have a strong imbalance in the relative sizes of ENCODE and Cistrome datasets, in favour of the latter.

### Filtering on high-signal improves the performance of location dependent machine learning algorithm

We now investigate the effect of filtering according to high signalValue binding sites (and where they match) on the Transcriptional Interaction and Coregulation Analyser (TICA). TICA attempts to find co-locating pairs of TFBS, and exploits the distribution of their distances to predict whether two Transcription Factors (TFs) are interacting.

We ran the TICA pipeline on all the different JOINs between ENCODE and Cistrome described in Methods. We used the default settings for the HepG2 cell line, as described in [10]. To validate our results, we used the following approach: consider the three interactions databases that we used to tabulate our list of accepted positives. For BioGRID and TRRUST, we extracted all the interactions reported that only mention eligible Transcription Factors (TFs) (i.e., those found in HepG2 in both databases). There is some nuance when dealing with CORUM, as it reports complexes of both Transcription Factors (TFs) and non Transcription Factors (TFs). Thus, we extracted all complexes that contains at least two eligible Transcription Factors (TFs), and consider two Transcription Factors (TFs) to be interacting if they are found in at least one of such complexes together. This results in a set of 29 validated interactions out of 435 possible ones (after reducing for symmetry and factoring in that TICA does not predict self interactions).

We evaluate the effect of the filtering by comparing recall, precision, and accuracy of the predictions, using the above reference set. Care should be taken as we define the above terms, because of two reasons:

1. The test set is unbalanced.
2. It is not self-evident what a negative case means in this situation. The fact that these databases do not report a particular interaction does not mean that there is none, but only that no evidence has been found yet. This is relevant in particular when considering “false positive”, as some of them are likely to be new, undetected interactions. For example, Forkhead Box A1 (FOXA1) and Forkhead Box A2 (FOXA2) are known to bind to “FOXA” sites and interact to regulate human Type I Iodothyronine Deiodinase (hDIO1) [11].

Keeping this in mind, we give the following definitions:

- A “validated” interaction as one where evidence is found in at least one of the databases above.
- A “validated prediction” is a positive TICA prediction that is also a validated interaction.
- Conversely, a “non validated” interaction is one where no evidence is found in any database, and a “non validated prediction” is a positive but non validated TICA prediction.

**Fig. 5:**
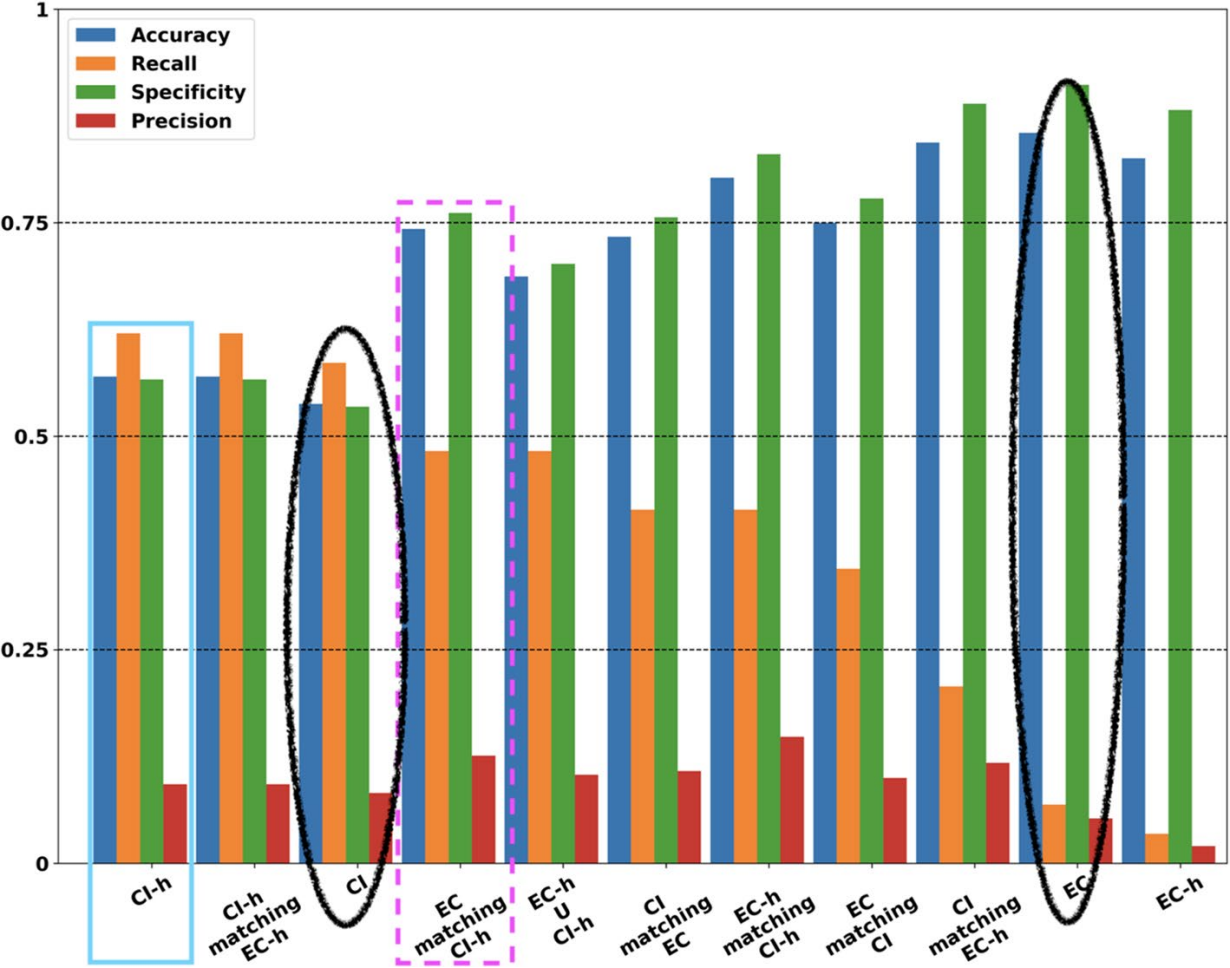
Performance measures of TICA predictor, using various combinations of ENCODE and Cistrome. *Notation:* EC-h stands for ENCODE-high, viz., those TFBS in ENCODE whose signal is above the 75th percentile; ditto for CI-h (Cistrome-high). Histograms sorted by recall. *Dashed rectangle:* recommended datasets for general data analysis; *solid rectangle:* best datasets for recall-dependent applications; *ovals:* ENCODE-full and Cistrome-full performances for comparisons (see main text for details)

In this framework, true positives are validated predictions, false positives are non validated predictions, false negatives are validated interactions that are not predicted as such, and true negatives are non validated interactions that are not predicted either. In Fig. 5 we show the performance metrics of TICA using the different database JOINs that we described in 5. The chart is sorted by recall. This is done because as we mentioned, our presumed reference contains very few positive cases.

While using Cistrome-high peaks (solid, cyan rectangle in Fig. 5) leads to the best recall, the best dataset to use is arguably ENCODE after filtering based on Cistrome-high (dashed, magenta rectangle in Fig. 5). This is because while this filtering achieves lower levels of recall (0.480 versus 0.620), it has much more specificity (0.760 versus 0.560). Using ENCODE-high matching Cistrome-high also yields good performance, with slightly lower recall (0.414) and higher specificity (0.830). Notably, there is a stark difference in performance between using only Cistrome and using only ENCODE (black ovals in Fig. 5). Such difference adds further evidence that our initial question is justified, and that the two data sets are not equivalent as they are.

**Fig. 6:**
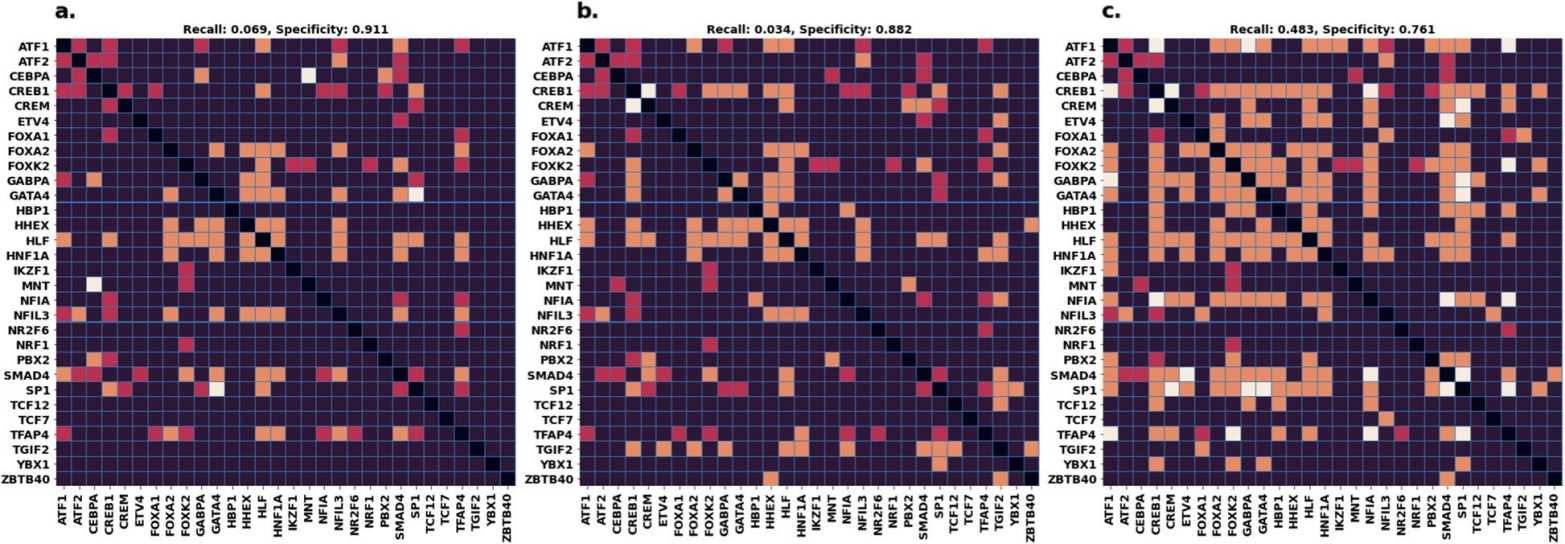
Validation matrices of TICA results using ENCODE data. **Legend.** *White:* validated POS; *dark red:* validated NEG; *orange:* non validated POS; *purple:* non validated NEG; *black:* NA. **a** Full ENCODE database. **b** ENCODE using only high signal binding sites. **c** ENCODE sites matching Cistrome-high sites

Finally, we show in Fig. 6 the effect of filtering on the prediction themselves. In particular, we compare the predictions of between using ENCODE (with no filters), ENCODE-high, and ENCODE matching Cistrome-high. Firstly, we observe that the majority of TF-TF combinations are reported as “true negatives” (i.e., non interactions). This is be expected, because a majority of proteins do not interact with each other [12]. Secondly, we observe that simply limiting ourselves to high signals in ENCODE is not sufficient to improve recall, but matching Cistrome high-signal peaks significantly improves it (from 2 to 14). This is a significant improvement over using ENCODE on its own. Consequently, we also note that the number of non-recalled interactions (“false negatives”) is reduced with the addition of Cistrome peaks (from 27 to 15). This is also quite good, because it means we can recover more of the existing biological network. Finally, the number of “false positives” (non validated predictions) increases (from 36 to 97). While some of these will be incorrect predictions, we speculate that a good amount of them will be predictions of novel co-operative TF-TF interactions,. Alternatively, some of these could be predictions of competitive TF-TF interactions (e.g. the earlier mentioned FOXA1-FOXA2 interaction [11]); this is because BioGRID, CORUM, and TTRUST could only be used to validate co-operative TF-TF interactions.

## Discussion

By considering only positional information of binding sites, one can already observe a nontrivial difference between ENCODE and Cistrome (Fig. 2). Part of this difference can be explained by the fact that Cistrome includes additional data sources, such as ROADMAP Epigenomics. In addition to this, the CistromeDB research group has chosen to add some lower-quality samples that nonetheless may include useful clues to some aspect of regulatory biology not represented by other samples in the database [13]. However, this still does not adequately explain the low fraction of regions that ENCODE can match (also Fig. 2). With an average percentage of sites recalled around 30 to 50% for both databases, it does not seem reasonable to the use the two data sources interchangeably.

The Cistrome research team has collated the raw data from all sources and reanalysed it using the ChiLin pipeline [13, 14]. This pipeline is subtly different from the standard ENCODE pipeline. In particular, sequences are aligned using the Burrows-Wheeler Aligner (BWA) in the former and bowtie2 in the latter; ChiLin preserves regions with high overlap with blacklist sites, while ENCODE does not; and ChiLin preserves 1 tag if multiple overlapping ones are found in a location, while ENCODE preserves all. The latter two points in particular could help explain the observed differences: blacklist areas [15] are sites that have shown consistent patterns of high signal and low binding affinity, independent of cell line. A difference in how these regions are implemented could lead to substantial differences in the reported binding events. The ChiLin pipeline also handles replicates in a different fashion, namely by calculating Pearson correlation of reads per million (wigCorrelate) and computing the percentage of overlapping peaks. This approach is quite different from ENCODE’s Irreprodubile Discovery Rate (IDR) method and is possibly a cause of the observed differences. Tellingly, the difference in magnitude and distribution of signalValues between the ENCODE and Cistrome samples are far larger than the observed differences of the same between samples coming from either ENCODE of Cistrome alone. In fact, samples from the same database tend to have quite similar values and distributions of signalValue, likely due to the fact that they are derived from the same pipeline (cf. Supplementary Materials, Figure S1).

This raises the question of whether it is reasonable or not to use the common locations between ENCODE and Cistrome. On a superficial level, it would seem reasonable to assume that binding sites that are supported by two databases are more likely to be real. While this is indeed the case, more nuance must be applied. If there is no correlation between the binding signal and the probability of matching, the distributions of the signal bins in Fig. 3 would behave roughly like the expected averages column. Clearly, experiments do not show this. On the contrary, there seems to be a tendency for matches to be between high or at most medium signal binding sites (Fig. 4). This is important for two reasons. First, it makes it statistically very improbable that there is no correlation between the levels of signalValue and the chance that the input sources are in agreement. Second, high quantities of tag reads detected at a location is typically considered evidence of binding events in that location [16]. Given that ChIP-Seq experiments involve assaying protein-DNA binding in vivo [17], and given that the target cell lines are immortalized [18] and thus essentially the same, it seems reasonable to assume the areas of highest binding should be consistent between the two databases. We tested whether binding sites from ENCODE and Cistrome are likely to be found in the blacklist / High-Occupancy Target (HOT) regions provided by [19]. Specifically, we counted the number of bases that exhibit a binding event from ENCODE and Cistrome and also overlap with a HOT region; we did this for different levels of signal, as well in the case where regions are matched (Supplementary Materials, Figure S9). We can see that when ENCODE and Cistrome are matched, the proportion of loci which overlap HOT region is rarely more than 5%, as opposed to the general case, and the presence of high signal slightly reduces this proportion. This support the theory that high signal-value area are sites of actual binding, made easier to detect by intersecting the two databases.

We turned to our previously published algorithm, TICA, to investigate whether binding sites filtered by signalValue improve the quality of downstream positional analysis. It is important to note that TICA does not require information about the signalValue, but nonetheless does include a reduction to 100bp windows around the peak summits [10]. This makes it a highly suitable candidate to test the effect of signalValue filtering.

The results of this analysis are quite telling. On the one hand, ENCODE datasets appear to employ protocols that are very stringent, being unable to capture some of the interesting biological information (i.e., only a recall of 7% at best). On the other, Cistrome datasets capture more of the interactions between transcriptions factors inscribed in ChIP-Seq datasets, but this comes with the cost of increasing the false alarm rate to levels that researchers may not be comfortable with (prediction heatmaps for Cistrome are reported in Supplementary Materials, Figure S8). Filtering ENCODE (resp., Cistrome) TFBS that match corresponding ones in Cistrome (ENCODE) improve the recall (in the case of ENCODE) and specificity (for Cistrome) of the predictions. While in theory this filtering reduces info and should lead to lower recall, in practice it doesn’t, so it is likely that most ENCODE peaks removed this way are noise peaks. However, we found that additional filtering by restricting putative matches to only those sites that have high signal lead to even better metrics for our predictor (cf. Fig. 5, dashed rectangle, Fig. 6). This suggests that restricting the input data to binding sites with high signalValue removes an additional set of noise peaks, which is in line with our assumption that TFBS with high signalValue are more likely to be real.

Finally, our initial hypothesis that ENCODE and Cistrome datasets are not directly comparable is supported by looking at the metrics for the two (cf. Fig. 5, ovals). While it may appear that Cistrome has the overall advantage, one should remember that the assumed reference is strongly imbalanced in favour of negative cases. Thus, having lower specificity will, in general, result in a large number of false positives added to the result set. We therefore recommend, as best practice, to use ENCODE as a base and filter it using Cistrome, as this combines the high recall of Cistrome with preserving a good amount of specificity from ENCODE (cf. Fig. 5, dashed rectangle, and Fig. 6).

We summarize below our main findings concerning the use of ENCODE and Cistrome TF binding signals and provide some guidelines for use in the context of machine learning pipelines:

1. ENCODE and Cistrome binding site “as is” datasets are far from optimal when used by themselves. Binding sites exhibit significant discrepancies in terms of positioning and signalValues, even among Transcription Factors (TFs) in the same cell line.
2. Under the assumption that high signalValue binding sites are more likely to be real, this discrepancies can be lessened. By ranking binding sites according to the magnitude of their signalValue, and extracting only those with high values, the resulting sets are much more likely to be overlapping.
3. If both ENCODE and Cistrome are available as input data for position-based algorithms, we recommend that ENCODE data is used after filtering for binding sites overlapping with Cistrome-high sites. This ensures the best compromise between quantity and quality of the binding sites.
4. However, if the ability to uncover existing information (as opposed to discarding artifact binding sites) is critical, using Cistrome as a base is recommended; filtering for high signalValue(s) provides better results.

## Conclusions

In this study, we set forth to investigate whether the signalValue attribute of Browser Extensible Data (BED) narrowPeak samples could be used to locate area of consistency between ENCODE and Cistrome datasets. We showed that a) the probability of matching two binding sites with high signalValue is significantly higher that expected, and b) if a binding site with high signal matches another, it is highly likely that the second one will have high signalValue as well. Finally, we used TICA to show that combining the two databases yields improved results, especially when filtering for high signalValue.

Our selection of high, medium, and low binding sites is based on the mediocrity principle (we assume the majority of binding sites will have “medium” values) and is the same for both databases. One could investigate whether the signalValue labeling is database-dependent. Figure 4 seems to suggest that the ENCODE high signalValue threshold should be relaxed beyond the top 25% percentile. Further research will be required to estimate an optimal threshold.

The 100bp size for peak summit windows was chosen to account for the natural uncertainty of narrowPeak binding sites [20] in a simple way. The wide range of overlaps shown in Fig. 2 suggests that it could potentially affect the overlap areas and thus the downstream analysis. Based on preliminary experiments, it appears that this phenomenon does not perturb our conclusions, but additional work could be done to estimate the optimal size of the window itself.

Finally, it is important to note that while our observations so far support these recommendations, one must take care when generalising them to an arbitrary machine learning algorithm. Investigators should make sure that their pipelines rest on similar hypotheses (namely, positional-based analysis based on binding site distances and not on the signalValue). Also, while false positive data can contain a significant number of true positives, the rest is still likely false positives and thus the precision value cannot be entirely discounted.

## Methods

### Data sources

We downloaded ChIP-Seq experiment data concerning human TFBS from both ENCODE and Cistrome (with permission). In the case of ENCODE, we limited ourselves to datasets processed using conservative IDR thresholding [1, 21]. Data format is BED narrowPeak. All datasets are aligned using the Genome Reference Consortium human build 38 (GRCh38). Data was downloaded from the relevant websites in March 2020. Data between different cell lines is not directly comparable due to differing biological conditions; thus, we limited the scope of the analysis to three cell lines: *GM12878* (lymphoblastoid), *HepG2* (liver carcinoma), and *K562* (myelogenous leukemia). We used the list provided in [22] to filter our protein list and keep only human Transcription Factors (TFs). Furthermore, we removed all Transcription Factors (TFs) that have significantly more samples in the datasets than others (due to being the object of more experiments). In our datasets, all Transcription Factors (TFs) except for 6 (CCCTC-binding Factor (CTCF) in HepG2, K562 and GM12878, RE1 Silencing Transcription Factor (REST) in GM12878, and NRF1, V-Myc Avian Myelocytomatosis Viral Oncogene Homolog (MYC), and GATA Binding Protein 1 (GATA1) in K562) have less than or exactly 9 samples. These are non tissue specific Transcription Factors (TFs) with general functions; we also note that many of these belong to the zinc-finger family. Thus, it is reasonable to impose an upper limit of 9 samples and exclude them. The reason for this is that those Transcription Factors (TFs) would skew our descriptive statistics down the line. The final datasets are described in Table 1.

**Table 1:** Descriptive statistics of TFBS datasets used in this study

Cell	Database	Number of TFs	Number of samples per TF	Median records per TF
HepG2	ENCODE	94	2	14737
	Cistrome	88	3	19711
GM12878	ENCODE	47	2 to 3	11193
	Cistrome	85	1 to 2	18083
K562	ENCODE	159	3 to 5	10429
	Cistrome	149	2 to 3	17377

Number of sample per TF reported as a range for improved legibility. Median is reported for the number of records per TF due to the presence of outliers

#### Signal filtering and sample merging

Before beginning the process of normalisation and comparison, we removed from our input all TF binding sites whose signalValue lies above the 90th percentile of the signalValue distribution. This was done because significant outliers in the right tail are unlikely to be actual biological phenomena as opposed to artifacts from the experiments. We show the effect of this filter in Supplementary material, Figure S1. Notably the Cistrome distribution do not change in shape after the filter, but we decided to do it anyway to preserve the ratios of regions found in the databases. This threshold is not a sensitive one: removing signalValues above the 85th or 95th percentile does not significantly alter the results (data not shown).

**Fig. 7:**
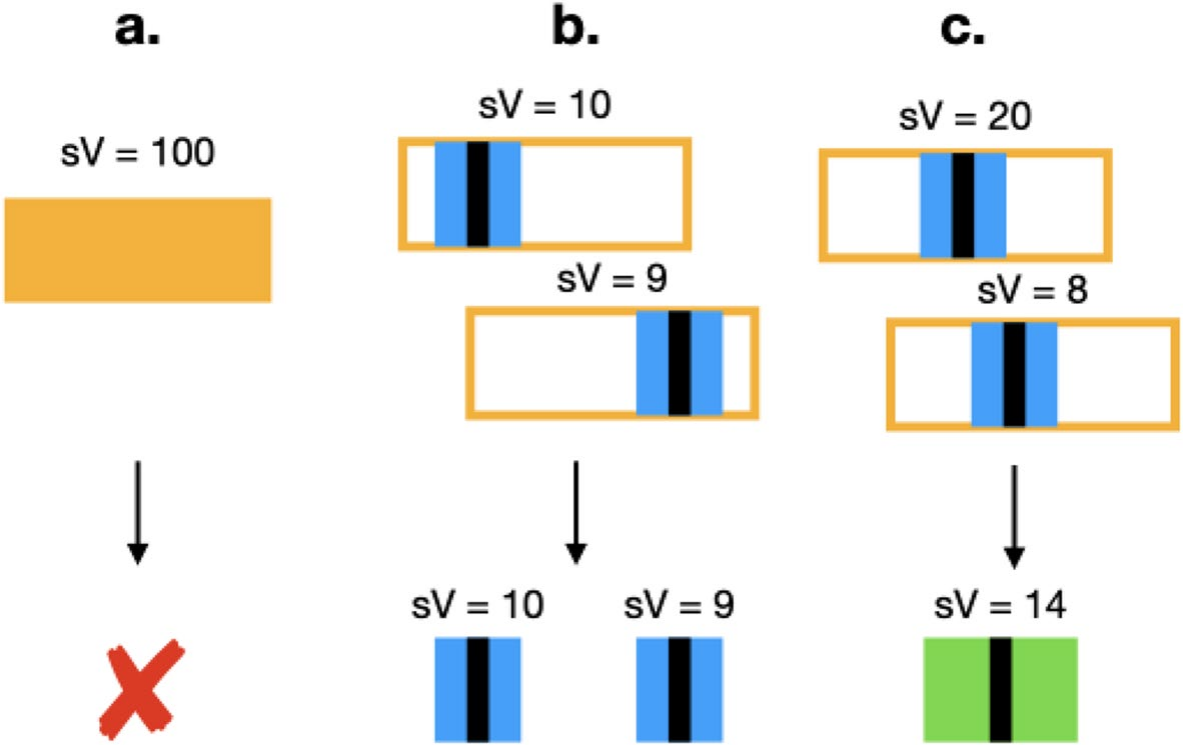
Various examples of how overlapping binding sites are processed. Legend: *sV* stands for *signalValue* associated with a peak. *Highlighted boxes:* original binding site extension. *Black inner boxes:* point sources. *Blue boxes:* 100bp windows around the pointed sources. **a** This binding sites is filtered at the beginning because its signal is above the 90% percentile. **b** After shrinking to 100bp windows, the two regions do not overlap, so they are retained. **c** An overlap is found, and a new binding site is returned as the union of the two. The new binding site is given a signal of 14 (average of 20 and 8) and a new peak summit that is the average of the original two

In order to effectively compare binding sites across databases, one must deal with signal duplication. If two binding sites in two different samples represent the same biological phenomenon, it is unwise to keep them both, as it would result in data duplication. To solve this, we merge overlapping binding sites belonging to different samples of the same TF. Two genomic regions are *overlapping* if they are co-located in at least 1 base pair. Each narrowPeak region comes a *peak* attribute (punctual location of highest signal, or point-source). The *peak* is a prime candidate for the initial base of the putative binding site, so it is desirable to retain this location. Moreover, it is possible for two binding sites to overlap over a large number of base-pairs while having distant peak summits. To simply merge two such peaks if they have any overlap would be unwise, as doing so would result in potentially large regions (400-600bps) and artificial peak summits. Therefore, we collected all TFBS of each TF (grouping by cell line) and shrank each binding site to a window of 100bp before and after the peak summit. The value of 100bp for the half window was chosen as a nominal value to approximate the uncertainty of narrowPeak peak summits [20]. Then, we merge overlapping regions into a new region which is the union of all base pairs occupied by either input, and we assign to the output the average signalValue of the inputs. The output region has a new peak summit equal to the average of the original peak summits. Regions that are not overlapping with others of the same TF are retained as they are. In Fig. 7, we describe this procedure in three difference cases. For simplicity, in the rest of the paper we shall refer to these 201bp-wide windows as “regions”.

### Dataset matching according to overlaps

Intuitively, one could say that ENCODE and Cistrome have a large amount of concordance if many of their regions overlap, and the overlapping areas have similar levels of signalValue. To test this, we designed a two-fold approach: first, we estimate the conditional Jaccard Indices of the regions that compose each dataset. This is done by dividing the combined size of all overlapping areas between regions of the same TF (one dataset from ENCODE, the other from Cistrome) by the total size of each contributing sample. The closer this number is to 1, the more the corresponding dataset is recalled by regions in the other. However, the Jaccard Index is known to be sensitive to dataset size [23]. Thus, we also implement the Alroy correction of the Forbes coefficient [9]. The Alroy-corrected Forbes coefficient is defined as follows:

1 $$\begin{aligned} \frac{a(n+\sqrt{n})}{(a+b)(a+c)+a\sqrt{n}+\frac{1}{2}bc} \end{aligned}$$

where *a* is the number of basepairs where both ENCODE and Cistrome report a peak, *b* is the number of basepairs where only ENCODE reports a peak, *c* is the same for Cistrome, and n=a+b+c. This is shown to be less sensitive to dataset size and more precise in detecting relationships between samples. Second, we designed a signalValue-wise method to estimate overlap, as follows. We split the signalValue distribution of the regions described in Signal filtering and sample merging section into three bins: low signal (bottom 25% of the distribution, denoted L), medium signal (between 25% and 75% of the distribution, denoted M), and high signal (upper 25% of the distribution, denoted H). The resulting bins have a 1:2:1 size ratio. We then ranked each region according to its signalValue. This is a form of normalization where we forget the actual value of the signal, and only consider the rank, making signalValues from ENCODE and Cistrome comparable. Finally, we intersected samples of binding sites for the same TF that belong to different databases (viz., ENCODE and Cistrome), looking for areas of overlap. We used the genometric JOIN operators (with the condition “overlap”) described in [24] to detect overlapping genomic regions. Each overlapping pair of regions defines a tuple of signalValue tags (i.e., (L,L), (L,H), and so on). The distribution of these will later be used to evaluate the consistency of signalValues between the databases.

All the joins performed are listed below. EC (“ENCODE-Full”) represents the entire set of ENCODE binding sites, and similarly for CI (“Cistrome-Full”).

- $a \bowtie _g b: a \in \{\text {EC-H}, \text {EC-M}, \text {EC-L}\}, b \in \{\text {CI-H}, \text {CI-M}, \text {CI-L}\}$, i.e., the nine symmetrical joins used to estimate the actual distribution of the pairs of signalValue tags;
- EC  CI, CI  EC-H, CI-H  EC-H, EC-H  CI-H, i.e., the left joins to check how many of the EC and EC-h peaks are confirmed by a CI-h peak (and viceversa);

where $\bowtie _g$ denotes the genometric JOIN described above and  the left outer genometric JOIN; EC-H stands for ENCODE-high, the set of all binding sites in ENCODE that are tagged with the high-signal (H) label. Similarly, we denote EC-M for ENCODE-medium, CI-L for Cistrome-low, and so on.

All extractions are performed by TF and by cell, viz., only binding sites that belong to the same TF in the same cell line are joined. Additionally, we limit this extraction only to those Transcription Factors (TFs) that are found in both databases and in the same cell line (viz., a TF is eligible if its ChIP-Seq samples are available both in ENCODE and Cistrome in the same cell line). This results in 30 eligible Transcription Factors (TFs) in HepG2, 19 eligible Transcription Factors (TFs) in GM12878, and 93 eligible Transcription Factors (TFs) in K562. The full joining pipeline is described in Supplementary Materials.

### Target metrics for database comparisons

There are many different types of machine learning algorithms and pipelines that process TFBS data. Many of them rely on a combination of: binding site positions on the chromosome [10, 25, 26]; statistical significance of the binding event, such as *p*-value or q-value [27, 28]; size of the binding site itself and position of its predicted peak summit [29].

Since our ultimate goal is to provide guidelines in the context of machine learning algorithms, we compared the two databases when used as input data for prediction pipelines. As an example, we used TICA, introduced in [10]. TICA is an algorithm and related web service that predicts whether two transcription factors interact (both co-operative and competitive interactions) based on the relative position of their closest binding sites. TICA relies on two reasonable assumptions: one, that most TF pairs do not interact; and two, that close range positioning between TFBS is a strong indicator of physical interaction. Given two candidate Transcription Factors (TFs), TICA extracts those pairs of TFBS (one from each) that are at minimal distance (named *minimal distance TFBS couples*) and computes their genomic distances, then compares their distance distribution to the null distribution of minimal distance TFBS couples of all possible TF-TF combinations in the same cell line. It predicts interactions based on *p*-value cutoffs on said distributions. Notably, TICA requires the input datasets to be provided in BED narrowPeak format, but does not employ the signalValue as a feature. Thus, we can estimate the effect of filtering based on signalValue without introducing bias in the result of the algorithm itself. To do so, we run the TICA pipeline using as input the various JOIN extractions described above. We evaluate the quality of the output by comparing it to three data sources:

- BioGRID v4.3 [30] (MultiValidated, Physical interactions only)
- CORUM [31]
- TRRUST [32]

While none of the above data source singles out TF-TF dimer pairs, each of them provides partial evidence of experimentally detected protein-protein interaction. In particular, TRRUST lists pairs of TF and regulation targets, and CORUM contains a list of protein complexes. We assume that evidence of protein-protein physical interaction between two TFs translate to evidence of direct co-operative TF-TF interaction. Thus, we built a positive set of “validated” interaction as the union of all interactions reported by any of the three sources. It should be noted that we are not training a prediction model in the classical sense, but rather evaluating the effect of differing data sources on an existing, pre-trained model. Thus, even though in the following we will use language typical of supervised machine learning methods (such as recall and precision), we do not need to split this validated set into training and testing sets.

### Software

Dataset joining was performed using the GMQL JOIN and MAP frameworks [24], implemented using Synchrony iterators [33]. TICA is available at www.gqml.eu/tica.

## Supplementary Information

Supplementary Material 1

## Data Availability

All datasets are publicly available for download from their respective repositories (ENCODE and Cistrome). TICA is available for testing at gmql.eu/tica.

## Abbreviations

BED: Browser Extensible Data
BWA: Burrows-Wheeler Aligner
ChIP-Seq: Chromatin ImmunoPrecipitation followed by Sequencing
CTCF: CCCTC-binding Factor
FOXA1: Forkhead Box A1
FOXA2: Forkhead Box A2
GATA1: GATA Binding Protein 1
GRCh38: Genome Reference Consortium human build 38
hDIO1: human Type I Iodothyronine Deiodinase
HOT: High-Occupancy Target
IDR: Irreprodubile Discovery Rate
MYC: V-Myc Avian Myelocytomatosis Viral Oncogene Homolog
NFIL3: Nuclear factor interleukin 3 regulated
NRF1: Nuclear Respiratory Factor 1
REST: RE1 Silencing Transcription Factor
SP1: Specificity Protein 1
TF: Transcription Factor
TFBS: Transcription Factor Binding Site
TICA: Transcriptional Interaction and Coregulation Analyser
