# Supplementary material for: A comparative analysis of ENCODE and Cistrome in the context of TF binding signal
Source: BMC Genomics. 2024 Aug 30;25(Suppl 3):817. doi: 10.1186/s12864-024-10668-6 (PMC11363379; doi:10.1186/s12864-024-10668-6)
Supplement: Supplementary file 1 — Supplementary Material 1 [file 12864_2024_10668_MOESM1_ESM.pdf]

A comparative analysis of ENCODE and Cistrome in the context of TF binding signal - Supplementary Materials

Stefano Perna, Pietro Pinoli, Stefano Ceri, Limsoon Wong

August 2022

Methods

Database intersection pipeline

To extract the joint signalValue distribution of ENCODE and Cistrome on a particular TF, we perform the following steps.

1. Remove all regions from each sample whose signalValue is in the top 10% of the distribution of their sample.

2. Shrink each binding site to a 201bp-wide region centered on the point-source. In particular, each region is altered to that its chromStart is now the original chromStart plus the peak (the point-source displace, an attribute of narrowPeaks) minus 100bp; and the new chromEnd is the original chromEnd plus peak, plus 100bp, plus one extra bp to account for the fact that bed files are 0-based.

3. For a given TF in either ENCODE or Cistrome (separately), merge all regions that overlap on at least 1 base pair. If a region does not overlap with any other, it is kept as is; if it does, all overlapping regions are merged, and the ouput region is given a new point-source equal to the average position of the input point-sources, and ditto for the signalValue. This has the additional effect of merging all samples for each TF into a single file, and eliminating replicates.

4. Each sample is binned according to its signalValue distribution. The lower 25% of the signalValue distribution is labelled as “L”, the upper 25% as “H”, and the remaining 50% as “M”.

At this point, samples are ready to be joined. Joins are performed TF-wise and cell-wise, viz., only samples of the same TF and in the same cell can be joined. In the following, fix one TF and one cell be fixed , such that there is only one sample from ENCODE and one from Cistrome.

There are two alternative ways to join them.

- If two bins have to be directly joined (say, for example, that ENCODE-high has to be joined with Cistrome-low), we use the Genometric JOIN operator from the GMQL language. The genometric JOIN returns all regions from the cartesian product of the samples such that they satisfy one or more conditions based on their distance. In our case, we use the "Overlap(1)" clause, which states that two regions are returned if they overlap on at least 1 base pair. The genometric JOIN requires an output clause as well, that states which part of the joined pair of regions is returned. We use the "Intersection" clause, which states that the output will be formed by the intersection of the joined region.

- If, on the other hand, one dataset has to be filtered using another one (for example, one wants all peaks from ENCODE-high that match at least one peak from Cistrome-high), we opted to use the genometric MAP instead. MAP returns all regions from the left operand (the "reference"), after performing a certain operation on every region (if any) of the right operand (the "experiment") that overlaps it. The default operation (which we employ) is COUNT, viz., the output contains all regions from the reference with a count of how many regions from the experiments it overlaps with. Regions with a COUNT equal to zero are filtered. This preserves the entirety of the left regions and is logically more consistent with that we want to achieve. In practice, though, the results are similar enough that a JOIN could be used instead.

Results

Cell line GM12878

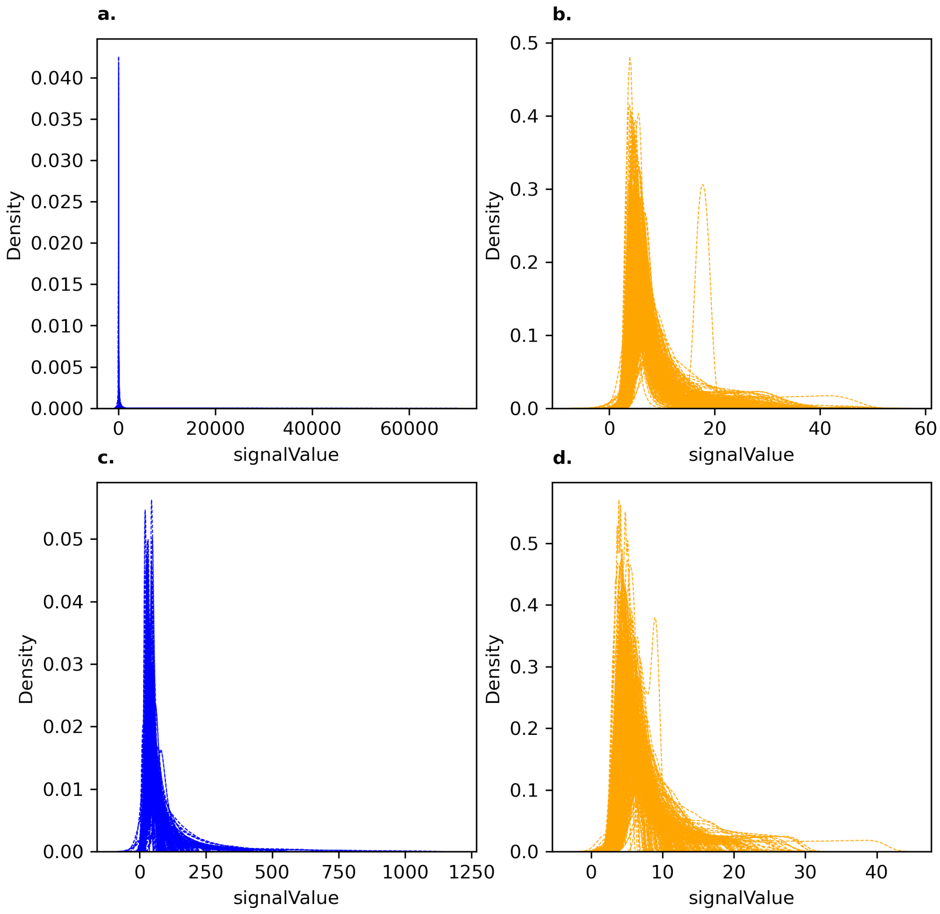

Figure 1: Effect of right tail filtering on ENCODE and Cistrome signalValue distributions. **a.** ENCODE full signalValue distribution. **c.** ENCODE distribution after removing the top 10% highest signalValues. **b.** and **d.** Ditto for CISTROME.

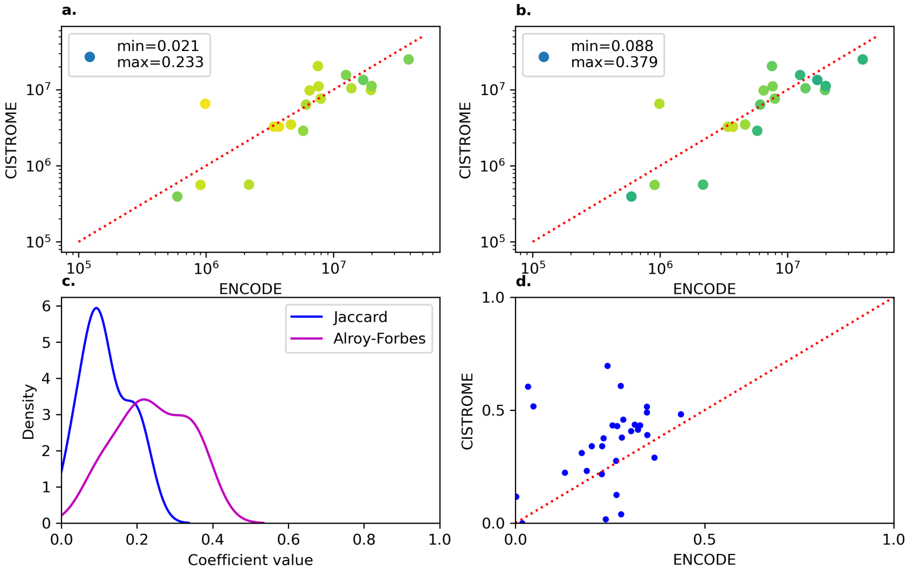

Figure 2: In all graphs, a dot represents a single TF. **a.** General JaccardIndex scatterplot for GM12878. *x axis:* number of basepairs occupied by all regions for this TF in ENCODE (log10) *y axis:* number of basepairs occupied by all regions for this TF in Cistrome (log10) *Color:* General JaccardIndex. Darker color represent higher values. **b.** Alroy-Forbes coefficient scatterplot for GM12878. *x axis:* number of basepairs occupied by all regions for this TF in ENCODE (log10) *y axis:* number of basepairs occupied by all regions for this TF in Cistrome (log10) *Color:* Alroy-corrected Forbes coefficient. Darker color represent higher values. **c.** Distribution of Jaccard Index and Alroy-corrected Forbes coefficient values in HepG2. Note that a value of 1 of the Alroy-Forbes coefficient denotes high-correlation. **d.** Conditional Jaccard Index scatterplots on GM12878. *x axis:* Conditional JI with respect to ENCODE. *y axis:* Conditional JaccardIndex with respect to Cistrome.

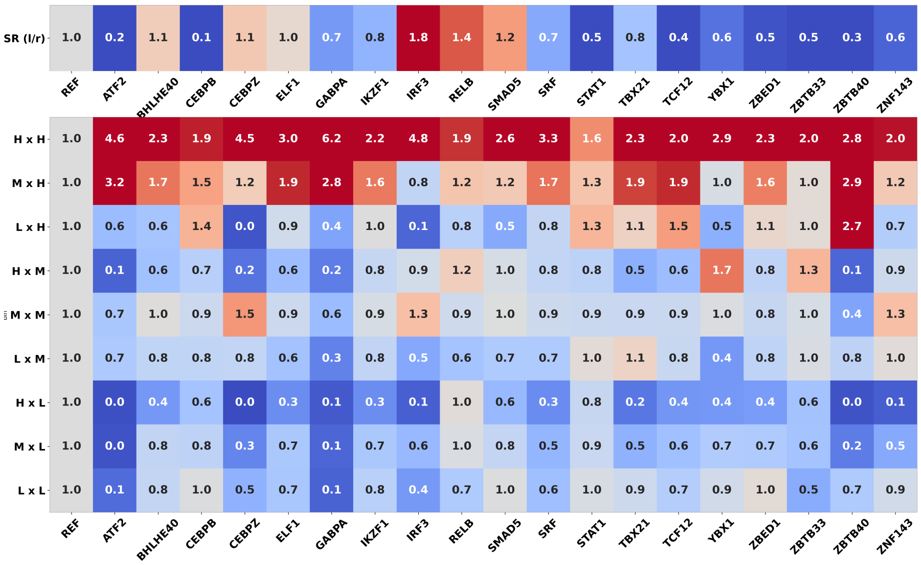

Figure 3: Heatmaps of the signal distribution among joined binding sites in GM12878. **Top row:** size ratio between ENCODE and Cistrome. A size ratio x means that the number of binding sites in ENCODE divided by the number of those in Cistrome equals x (red: ENCODE has more binding sites, blue: Cistrome has more). **Bottom rows:** for each TF (columns), ratio of joined binding sites that fall into each bin compared to the expected null distribution under independence assumption. *Red:* bin is over-represented compared to the null; *blue:* bins is underrepresented.

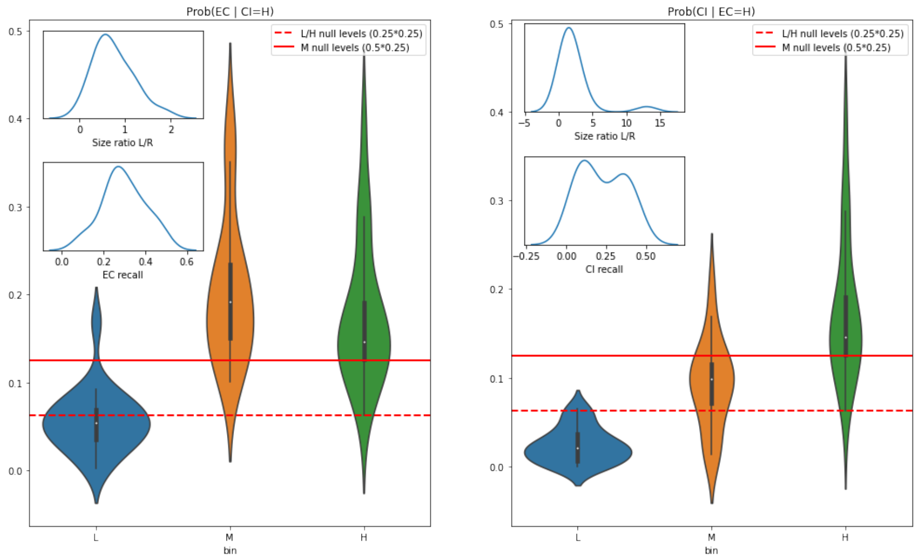

Figure 4: **a.** Conditional probability of ENCODE signal when matched with Cistrome-high binding sites in cell line GM12878. Red Lines: theoretical expected values assuming signal is independent of matching. Inset graphs: top - distribution of the size ratio between ENCODE sites and Cistrome sites among all TFs; bottom - distribution of the percentage of ENCODE sites recalled by Cistrome among all TFs. **b.** Conditional probability of Cistrome signal when matched with ENCODE-high binding sites. Red Lines: theoretical expected values assuming signal is independent of matching. Inset graphs: as left, with reverse databases.

Cell line K562

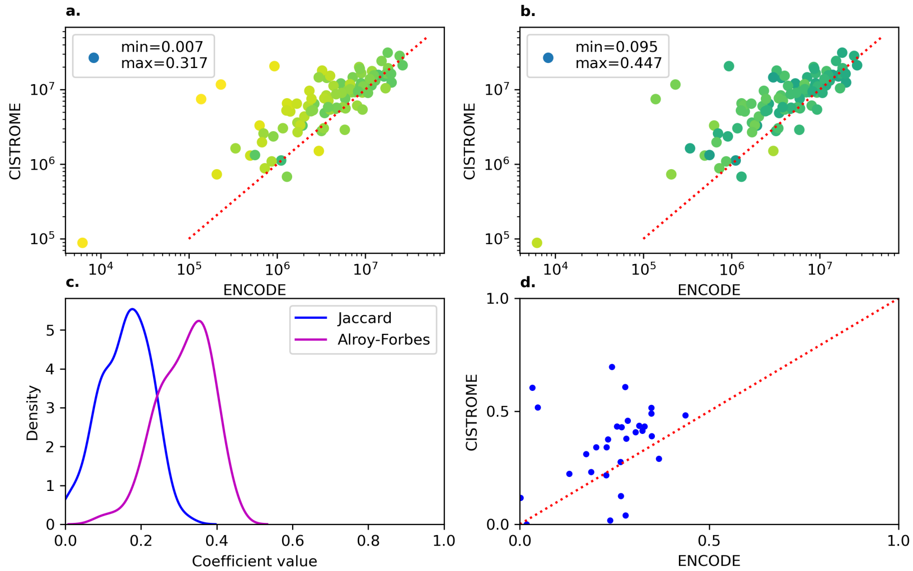

Figure 5: In all graphs, a dot represents a single TF. **a.** General JaccardIndex scatterplot for K562. *x axis:* number of basepairs occupied by all regions for this TF in ENCODE (log10) *y axis:* number of basepairs occupied by all regions for this TF in Cistrome (log10) *Color:* General JaccardIndex. Darker color represent higher values. **b.** Alroy-Forbes coefficient scatterplot for K562. *x axis:* number of basepairs occupied by all regions for this TF in ENCODE (log10) *y axis:* number of basepairs occupied by all regions for this TF in Cistrome (log10) *Color:* Alroy-corrected Forbes coefficient. Darker color represent higher values. **c.** Distribution of Jaccard Index and Alroy-corrected Forbes coefficient values in HepG2. Note that a value of 1 of the Alroy-Forbes coefficient denotes high-correlation. **d.** Conditional Jaccard Index scatterplots on K562. *x axis:* Conditional JI with respect to ENCODE. *y axis:* Conditional JaccardIndex with respect to Cistrome.

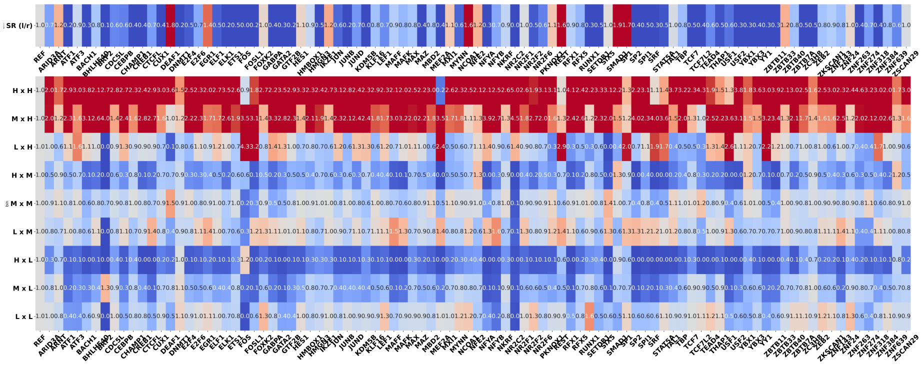

Figure 6: Heatmaps of the signal distribution among joined binding sites in K562. **Top row:** size ratio between ENCODE and Cistrome. A size ratio x means that the number of binding sites in ENCODE divided by the number of those in Cistrome equals x (red: ENCODE has more binding sites, blue: Cistrome has more). **Bottom rows:** for each TF (columns), ratio of joined binding sites that fall into each bin compared to the expected null distribution under independence assumption. *Red:* bin is over-represented compared to the null; *blue:* bins is underrepresented.

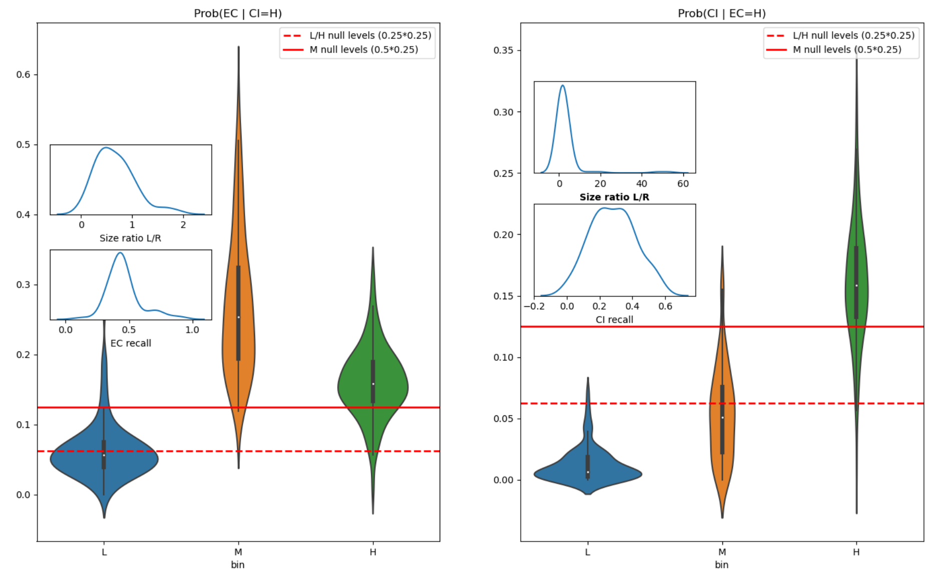

Figure 7: **a.** Conditional probability of ENCODE signal when matched with Cistrome-high binding sites in cell line K562. Red Lines: theoretical expected values assuming signal is independent of matching. Inset graphs: top - distribution of the size ratio between ENCODE sites and Cistrome sites among all TFs; bottom - distribution of the percentage of ENCODE sites recalled by Cistrome among all TFs. **b.** Conditional probability of Cistrome signal when matched with ENCODE-high binding sites. Red Lines: theoretical expected values assuming signal is independent of matching. Inset graphs: as left, with reverse databases.

Cistrome validation matrices on HepG2

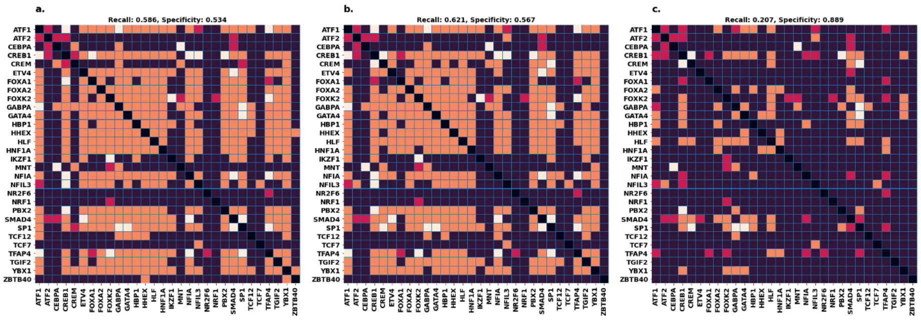

Figure 8: Validation matrices of TICA results using Cistrome data on HepG2. **Legend.** *White:* validated POS; *dark red:* validated NEG; *orange:* non-validated POS; *purple:* non-validated NEG; *black:* NA. **a.** Full Cistrome database. **b.** Cistrome using only high-signal binding sites. **c.** Cistrome sites matching ENCODE-high sites.

Analysis of HOT regions

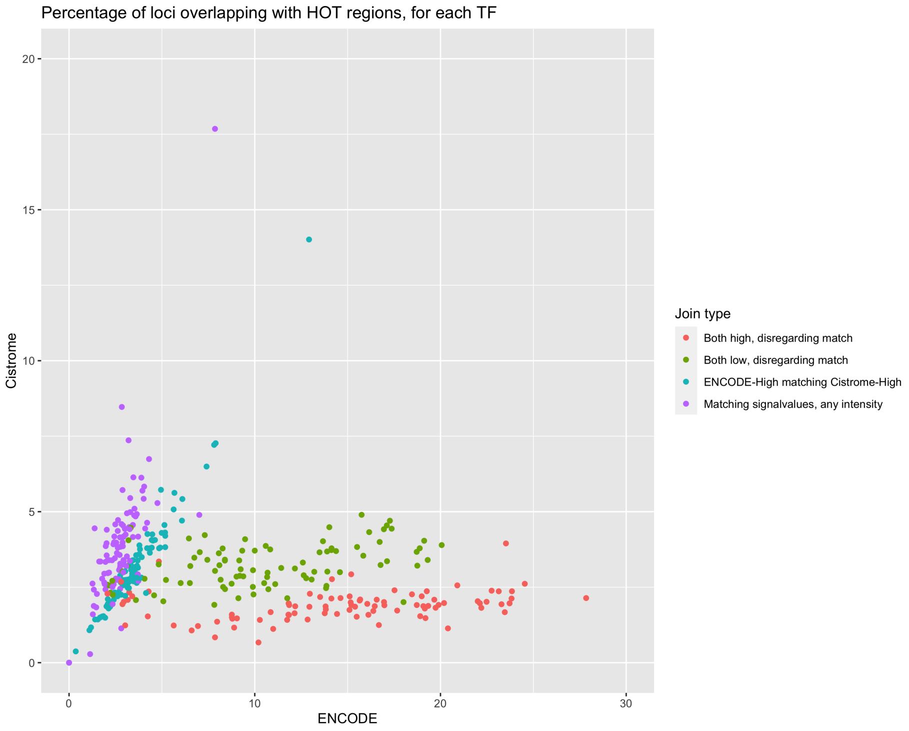

Figure 9: Analysis of the overlap between ENCODE and Cistrome regione with HOT (high-occupancy) regions, according to signalvalue and match. Each dot is (all binding sites of) a single transcription factor. x- and y-axes scaled to percentage of loci found to be overlapping with HOT regions.
